# Themes Depicted in Top-grossing Rated-R Films Released from 2012 to 2017

**DOI:** 10.7759/cureus.6844

**Published:** 2020-02-01

**Authors:** Andrew Watts, Justin Loloi, Kaila Lessner, Marina Mizell, Katherine Shoemaker, Tonya S King, Robert Olympia

**Affiliations:** 1 Emergency Medicine and Pediatrics, Penn State Hershey College of Medicine, Hershey, USA; 2 Internal Medicine, Penn State Hershey Medical Center, Hershey, USA; 3 Emergency Medicine, Penn State College of Medicine, Hershey, USA; 4 Epidemiology and Public Health, Penn State College of Medicine, Hershey, USA; 5 Emergency Medicine and Pediatrics, Penn State Hershey Medical Center, Hershey, USA

**Keywords:** rated-r, mediation, development, media, co-viewing, films, violence, themes

## Abstract

Introduction: Films continue to be a popular form of entertainment amongst children and adolescents. The objective of this study was to determine the most common themes depicted in a select number of rated-R films released 2012-2017.

Methods: A total of 25 films were independently viewed and analyzed by five reviewers.

Results: The most common positive themes were “encouragement”, “helping others”, “teamwork”, “compassion”, and “honesty” (2.3, 1.7, 1.7, 1.6, 1.4 mean events per hour, respectively). The most common negative themes were “use of inappropriate language”, “use of a lethal weapon”, “physical fighting”, “dishonesty”, and “demonstrating excessive anger” (8.9, 4.4, 3.4, 2.3, 2.2 mean events per hour, respectively).

Conclusion: The most common positive themes in our sample were associated with service, collaboration, and honesty/humility, and the most common negative themes were associated with violence. We encourage co-viewing and active mediation, focusing on the themes found in films, as a method to guide children through their development process.

## Introduction

Films represent a popular form of entertainment amongst children and adolescents. Over the last several years, there has been a significant rise in media consumption in the pediatric population, particularly in the realm of television and film. Studies have shown that children younger than eight years of age view approximately one-hour and forty-minutes of DVDs or television each day, while older children view almost four hours of content each day, and spend up to 28 hours per week in front of the television [1-2].

Given the considerable amount of time children dedicate to media consumption, there are strong opinions that television and film play a pivotal role in a child’s psychosocial development, both positively and negatively. Published studies have shown that the content of visual media may promote positive thoughts and behaviors, such as sharing, improved social interactions, racial attitudes, cooperation and empathy [3]. Conversely, several studies show that media violence exposure may lead to the development of deleterious behaviors such as aggression, bullying, antisocial attitudes and other forms of violence [4-6]. 

An important consideration is the viewing of rated-R films (“restricted - under 17 years old requires accompanying parent or adult guardian” defined by Motion Picture Association of America film rating system (www.mpaa.org)) by the pediatric population. Recently, there has been a significant rise in the pediatric viewership of rated-R films. Approximately three million adolescents in the United States aged 10 to 14 years old have watched rated-R films [7]. This is a major concern as children and adolescents may not always have parental permission to view these films. In this era of online streaming, video on-demand, and Digital Video Disc, children and adolescents may have access and exposure to films they would otherwise not be allowed to view. Studies have highlighted the importance of parental restriction and limiting a child’s access to rated-R films to mitigate the risk of negative behaviors such as smoking and early onset alcohol abuse [8-10].

Although there have been a few recently published studies examining the thematic content in top-grossing films released from 2005 to 2015 (limited to general audience (G), parental guidance (PG), and PG-13 rated films) and superhero-based films (excluding rated-R films), there have been no studies analyzing the content of top-grossing rated-R films that children and adolescents may have access to [11-12]. The objective of this study is to determine the positive and negative themes depicted in a select number of top-grossing rated-R films released from 2012 to 2017, and to compare the event rates between film genres. By identifying positive and negative themes found in rated-R films, pediatric healthcare providers and parents may use this information to develop co-viewing strategies to help in the education and development of children and adolescents who watch rated-R films.

## Materials and methods

We examined the positive and negative themes depicted in a select number of rated-R films released between 2012 and 2017. Our sample of 25 rated-R films were selected based on the highest lifetime gross profits reported by www.boxofficemojo.com on 2/1/2018. Included films required availability from either streaming media, video-on-demand, or Digital Video Disc. Film genre (action adventure, action comedy, comedy, drama and thriller/mystery), as assigned by www.IMDB.com, was determined for each included film.

The authors developed a data collection instrument that was composed of a predetermined list of positive and negative themes adapted from two previously published studies [11-12]. The data collection instrument enabled each of the five reviewers (AW, JL, KL, MM, KS) to independently document each event associated with a theme. For the purpose of this study, events were defined as actions or discussions, either stated directly in the film script or implied during a scene. Specifically, actions performed in the film and then later referenced were coded only at the initial encounter. We included verbal and non-verbal forms of praise/encouragement and discouragement/ridicule/taunting for both positive and negative interactions. Prior to viewing the study films, certain coding guidelines were determined. Additionally, the authors developed a coding algorithm for use when more than one theme applied to a certain scene in a film. Following viewing, the completed data collection instruments were submitted to the primary investigator (AW) and the data was entered into Excel. 

Mean events per hour for each theme was determined using repeated measures Poisson regression models in SAS version 9.4, and were reported with corresponding 95% confidence intervals. The rates and confidence intervals for each film genre and year of release were estimated by entering these factors into the models separately, with adjustment for reviewer. Individual positive and negative themes were then analyzed following the same procedure described for the total positive and negative themes. This study was deemed exempt by The Institutional Review Board at The Pennsylvania State Hershey Medical Center.

## Results

Table 1 describes the rated-R films included in our study, noting the dominant positive and negative themes for each film. For all included films, the median year of release was 2014 and the mean lifetime gross profit was $180.76 million. Included films were stratified into five different genres: action adventure (n=6, 24%), action comedy (n=5, 20%), comedy (n=4, 16%), drama (n=5, 20%) and thriller/mystery (n=5, 20%). There was no significant linear relationship between the year of release and total positive themes (p= 0.89) or the total negative themes (p= 0.93). There were twice as many negative themes than positive themes, with the average rate of positive and negative themes being 27.0 (95% CI 24.5- 29.7) and 57.8 (95% CI 50.8- 65.6) events per hour for all films, respectively.

**Table 1:** Description of the rated-R films included in the analysis

Film	Year Released	Genre	Running Time (Minutes)	Lifetime Gross ($ Million)^b^	Dominant Positive Theme ^c^	Dominant Negative Theme ^c^
21 Jump Street	2012	Action comedy	110	139	Encouragement and support from friend/peer/co-worker	Use of inappropriate language/cursing in a malicious way
22 Jump Street	2014	Action comedy	112	192	Encouragement and support from friend/peer/co-worker	Use of inappropriate language/cursing in a malicious way
50 Shades of Grey	2015	Drama	129	166	Demonstrating honesty	Use of inappropriate language/cursing in a malicious way
American Hustle	2013	Drama	138	150	Demonstrating honesty	Use of inappropriate language/cursing in a malicious way
American Sniper	2014	Action adventure	133	350	Importance of teamwork	Use of a lethal weapon (gun, knife, etc.)
Argo	2012	Drama	130	136	Asking for help/Offering to help someone	Use of inappropriate language/cursing in a malicious way
Deadpool	2016	Action comedy	109	363	Encouragement and support from friend/peer/co-worker	Use of inappropriate language/cursing in a malicious way
Django Unchained	2012	Action adventure	165	163	Being confident in oneself, brave or prideful	Use of racial slurs
Get Out	2017	Thriller/Mystery	104	176	Encouragement and support from significant other	Use of inappropriate language/cursing in a malicious way
Gone Girl	2014	Thriller/Mystery	149	168	Importance of training/practice, planning and preparation, goal-setting	Use of inappropriate language/cursing in a malicious way
Identity Thief	2013	Comedy	112	135	Demonstrating compassion, empathy, humility or forgiveness	Use of inappropriate language/cursing in a malicious way
It	2017	Thriller/Mystery	135	327	Encouragement and support from friend/peer/co-worker	Use of inappropriate language/cursing in a malicious way
Kingsman: The Secret Service	2015	Action comedy	129	128	Importance of training/practice, planning and preparation, goal-setting	Use of inappropriate language/cursing in a malicious way
Logan	2017	Action adventure	141	226	Asking for help/Offering to help someone	Use of inappropriate language/cursing in a malicious way
Lucy	2014	Action adventure	90	127	Asking for help/Offering to help someone	Use of a lethal weapon (gun, knife, etc.)
Mad Max: Fury Road	2015	Action adventure	120	154	Demonstrating generosity/not being greedy or selfish/self-sacrifice	Use of a lethal weapon (gun, knife, etc.)
Neighbors	2014	Comedy	97	150	Encouragement and support from significant other	Use of inappropriate language/cursing in a malicious way
Prometheus	2012	Thriller/Mystery	124	126	Importance of teamwork	Use of inappropriate language/cursing in a malicious way
Silver Linings Playbook	2012	Drama	122	132	Encouragement and support from friend/peer/co-worker	Use of inappropriate language/cursing in a malicious way
Straight out of Compton	2015	Drama	167	161	Encouragement and support from friend/peer/co-worker	Use of inappropriate language/cursing in a malicious way
Ted	2012	Comedy	112	219	Encouragement and support from friend/peer/co-worker	Use of inappropriate language/cursing in a malicious way
The Conjuring	2013	Thriller/Mystery	112	137	Importance of having faith/higher power/religion	Demonstrating excessive anger/intimidation/bullying
The Heat	2013	Action comedy	123	160	Encouragement and support from friend/peer/co-worker	Use of inappropriate language/cursing in a malicious way
The Revenant	2015	Action adventure	156	184	Overcoming obstacles/ learning from mistakes/giving 110%	Use of a lethal weapon (gun, knife, etc.)
We’re the Millers	2013	Comedy	118	150	Encouragement and support from friend/peer/co-worker	Use of inappropriate language/cursing in a malicious way

Tables 2-3 show the total as well as the most common positive and negative themes depicted in our sample of rated-R films, then stratified by film genre, respectively.

**Table 2:** Most common positive themes (reported as mean events per hour [95% confidence interval]) depicted in our sample of rated-R films, then stratified by genre

Themes	All included films (N=25)	Action Adventure	Action Comedy	Comedy	Drama	Thriller/ Mystery	p-value across genres
Encouragement and support from friend/peer/co-worker	2.30 [1.78-2.98]	1.55 [0.91-2.65]	4.24 [3.00-5.93]	2.86 [1.81-4.50]	2.22 [1.26-3.92]	1.16 [0.58-2.33]	0.004
Asking for help/Offering to help someone	1.69 [1.43-2.01]	2.21 [1.57-3.13]	1.36 [1.07-1.73]	1.91 [1.41-2.59]	1.43 [0.98-2.08]	1.49 [1.17-1.91]	0.13
Importance of teamwork	1.66 [1.31-2.10]	2.26 [1.57-3.25]	2.60 [2.00-3.37]	1.43 [0.95-2.15]	0.84 [0.47-1.49]	1.06 [0.61-1.83]	<0.001
Demonstrating compassion, empathy, humility or forgiveness	1.57 [1.31-1.87]	1.94 [1.56-2.41]	1.58 [1.03-2.42]	2.30 [1.92-2.73]	1.14 [0.95-1.36]	1.04 [0.76-1.41]	<0.001
Demonstrating honesty	1.38 [1.09-1.75]	0.90 [0.69-1.17]	1.57 [1.31-1.89]	1.96 [1.37-2.81]	1.89 [1.10-3.25]	0.87 [0.60-1.26]	<0.001
Being confident in oneself, brave or prideful	1.26 [1.07-1.49]	1.30 [0.90-1.87]	1.37 [1.04-1.82]	1.04 [0.71-1.52]	1.52 [1.23-1.87]	0.99 [0.68-1.45]	0.238
Demonstrating loyalty	1.18 [0.97-1.43]	1.28 [0.88-1.85]	1.41 [1.02-1.95]	1.57 [1.24-2.01]	0.98 [0.84-1.13]	0.78 [0.45-1.33]	0.004
Overcoming obstacles/ learning from mistakes/giving 110%	1.08 [0.84-1.38]	1.60 [1.06-2.41]	1.17 [0.68-1.68]	1.01 [0.68-1.49]	0.83 [0.63-1.07]	0.64 [0.39-1.06]	0.011
Importance of training/practice, planning and preparation, goal-setting	1.05 [0.79-1.38]	0.95 [0.72-1.25]	1.54 [0.97-2.45]	0.83 [0.56-1.23]	1.09 [0.74-1.59]	0.81 [0.40-1.67]	0.19
Strong female characters	0.92 [0.60-1.12]	0.76 [0.38-1.49]	1.13 [0.59-2.17]	0.63 [0.29-1.38]	0.77 [0.44-1.37]	0.81 [0.57-1.15]	0.81

**Table 3:** Most common negative themes (reported as mean events per hour [95% confidence interval]) depicted in our sample of rated-R films, then stratified by genre

Themes	All included films (N=25)	Action Adventure	Action Comedy	Comedy	Drama	Thriller/ Mystery	p-value across genres
Use of inappropriate language/cursing in a malicious way	8.88 [7.19-10.97]	5.98 [3.46-10.36]	14.42 [13.50-15.41]	12.39 [10.39-14.77]	8.41 [5.77-12.28]	5.51 [3.52-8.61]	<0.001
Use of a lethal weapon (gun, knife, etc.)	4.39 [3.21-6.01]	8.23 [6.50-10.43]	7.83 [5.34-11.47]	1.46 [0.61-3.50]	1.44 [0.67-3.07]	1.52 [0.77-3.01]	<0.001
Fighting (physically)	3.37 [2.66-4.26]	4.34 [2.97-6.34]	5.41 [3.53-8.30]	2.74 [2.25-3.32]	2.19 [1.31-3.65]	1.95 [1.37-2.76]	<0.001
Lying/dishonesty/keeping secrets	2.27 [1.74-2.96]	1.37 [1.01-1.86]	3.05 [2.41-3.85]	3.29 [1.98-5.46]	2.25 [1.40-3.61]	2.01 [0.82-4.90]	0.002
Demonstrating excessive anger/intimidation/bullying	2.24 [1.78-2.82]	2.45 [1.88-3.19]	3.30 [2.50-4.37]	1.71 [1.37-2.13]	1.61 [0.82-3.16]	2.05 [0.92-4.53]	0.003
Alcohol use/abuse	2.18 [1.63-2.92]	1.08 [0.55-2.13]	2.90 [1.96-4.29]	3.53 [1.86-6.69]	3.12 [2.07-4.69]	0.93 [0.34-2.55]	0.016
Fighting (verbal abuse/arguing)	2.14 [1.67-2.73]	1.70 [1.02-2.82]	2.60 [1.95-3.48]	2.84 [2.12-3.81]	2.74 [1.79-4.19]	1.12 [0.70-1.77]	0.004
Murder (<4 people)	2.12 [1.40-3.20]	4.54 [3.12-6.60]	3.40 [1.83-6.32]	0.06 [0.01-0.34]	0.24 [0.08-0.73]	1.30 [0.70-2.42]	<0.001
Reference to pornography or sexual acts	1.90 [1.20-2.99]	0.45 [0.17-1.20]	3.31 [1.61-6.78]	4.36 [2.45-7.77]	1.88 [0.98-3.62]	0.72 [0.40-1.29]	<0.001
Discouragement/ridicule/taunting by friend/peer/co-worker	1.61 [1.22-2.11]	0.53 [0.34-0.83]	3.17 [2.20-4.57]	2.67 [1.90-3.76]	1.19 [0.82-1.74]	1.25 [0.59-2.63]	<0.001

There was a significant difference among the five film genres with respect to the rate of total positive themes (p<0.001), with adjustment for the variability among the reviewers. The thriller/ mystery films had a significantly lower rate of positive themes than the other four genres as seen in Table 2, and the drama films had a significantly lower rate of positive themes than the action comedy movies (p=0.007).

There was a significant difference among the five film genres with respect to the rate of total negative themes (p<0.001), with adjustment for the variability among the reviewers. The action comedy and comedy films had significantly higher rates of negative themes than the other three genres as seen in Table 3, but these two genres were not significantly different from each other (action comedy vs. comedy p= 0.09).

Overall, the repeated measures Poisson regression models for the total number of both positive and negative themes per hour found a statistically significant amount of inter-rater variability among the reviewers (p< 0.001 in each of the models).

## Discussion

Based on our sample of rated-R films released between 2012 and 2017, we demonstrate that there were twice as many negative themes than positive themes depicted. The most common positive themes were associated with service, collaboration, and honesty/humility, and the most common negative themes were associated with violence. These themes are similar to those depicted in top-grossing rated-G, PG, and PG-13 films released between 2005 and 2015; in fact, there was a 60% overlap of the ten most common positive themes and a 50% overlap of the ten most common negative themes when comparing these two film rating category groupings [11]. Common positive themes found specifically in our rated-R film sample, and not commonly found in the top-grossing rated-G, PG, and PG-13 study, included “demonstrating compassion, empathy, humility, or forgiveness”, “demonstrating honest”, “being confident in oneself, brave, or prideful”, and “demonstrating loyalty”. Common negative themes found in our rated-R film sample, and not commonly found in the top-grossing rated-G, PG, and PG-13 study, included “lying/dishonesty/keeping secrets”, “alcohol use/abuse”, “murder (<4 victims”), “reference to pornography or sexual acts”, and “discouragement/ridicule/taunting by friend/peer/co-worker”.

The pediatric population may be particularly susceptible to media influences and the varied themes they are exposed to in films. This influence may become more significant with the increase in media access, especially to restricted material in rated-R films. Expectedly, parents, guardians, and health care providers may play significant roles in not only the development of children but also their perception of what they view on the screen. It is especially important for parents and guardians to monitor what their children view and ensure the content is age-appropriate. If and when children start watching rated-R films, parents and guardians should engage them with questions to encourage them to critically think and analyze what they saw. Discussions on these topics can help children begin to differentiate between fantasy and reality, especially as it pertains to sex and violence [13].

Rated-R films may be problematic for children and adolescents, as negative themes can be depicted up to twice as often compared to positive themes, as evidenced by our study. These negative themes mainly portray violence (use of lethal weapons, fighting, demonstrating excessive anger/intimidation/bullying, arguing, and murder), inappropriate language/cursing, and substance use/abuse, all of which, should not be viewed by children. Exposure to violent themes, including the use of lethal weapons or murder, can lead to the development of non-ideal behaviors in children and adolescents, such as aggression, bullying, antisocial attitudes and other forms of violence [4-6]. Furthermore, several studies have demonstrated that exposure to rated-R films may be predictive of earlier alcohol use and earlier initiation of smoking cigarettes and marijuana [9,14-16]. Interestingly, parental restrictions on rated-R film viewing appear to have a significant impact on substance use in adolescents, with restriction leading to a lower susceptibility to cigarette smoking and a delay in marijuana and alcohol use initiation [14-15]. 

While rated-R films are often dominated by negative themes, they do also contain positive messages that have lasting beneficial influences on prosocial behavior and thought development [3]. Complex film storylines expose the viewers to complicated conflicts and circumstances and demonstrate how to navigate and respond to those situations [17]. Additionally, it has been shown that positive messages portrayed by the media can promote positive social interactions, improve racial attitudes, and influence the perspective of gender-based roles in our society [3,18]. Children who are frequently exposed to positive themes may learn important life lessons from characters’ situations and then translate these lessons to their own lives. In our sample of rated-R films, the most common positive themes were “encouragement”, “helping others”, “teamwork”, “compassion”, and “honesty”. These themes should be stressed during critical ages when children are learning to interact and developing relationships with both members of their family and those outside of the family unit. 

It is unrealistic to believe that children and adolescents will not be exposed to rated-R films due to availability of these films on video-sharing online websites or streaming on television or mobile electronic devices. Therefore, it is important for pediatric health care providers and parents/guardians to be aware of the content of rated-R film and to understand the impact that positive and negative themes have on the children and adolescents who view them. A method to optimize the development of children and adolescents who view rated-R films involves co-viewing these movies as a family and active mediation. Co-viewing and active mediation occurs when the parent or guardian discusses what it is being watched, either during or following the film. This method encourages the development of critical thinking and internally regulated values, and has been shown to decrease aggressive behavior, substance use, and early sexual behavior in adolescents [19]. While pediatric health care providers frequently recommend restriction and monitoring of media time in general, as well the avoidance of rated-R films, the promotion of co-viewing and active mediation would allow parents/guardians to be involved in the development of their children. A template for co-viewing and active mediation of rated-R films (“R-FILMS/POSITIVE/NEGATIVE”), based on the most common positive and negative themes found in our sample of top-grossing rated-R films, and adapted from the study of most common themes found in a sample of top-grossing films rated-G/PG/PG-13, can be seen in Table 4 [11].

**Table 4:** Template for co-viewing rated-R films with children and adolescents

R	Recreational drugs and alcohol usage	Give an example of a character who used drugs or alcohol, and were there any negative consequences to its usage. Have you ever been pressured to use drugs or alcohol? What should you do if faced with this situation?
F	Forgiveness, compassion, humility	What character demonstrated forgiveness, compassion or humility? Give an example in your life when you demonstrated forgiveness, compassion, or humility, and how did this make you feel? Have you ever held a grudge, and how did this make you feel?
I	Integrity and honesty	Give an example of a character who demonstrated honesty. Why is it important to be honest? In your life, have you ever been dishonest? How did it make you feel? Did you resolve your dishonesty?
L	Loyalty	Give an example of a character who demonstrated loyalty, and a character who stabbed another character in the back. Who have you demonstrated loyalty towards? Have you ever shown disloyalty towards another, and why did you do this?
M	Murder	Were there any examples of murder in the film? Why did the murder occur, and how else could the character have handled the situation?
S	Sexuality and pornography	Give an example of unhealthy sexual behavior or pornography in the film. Have you ever been in the situation when a sexual act was forced upon you? How did you handle this situation?
P	Preparation/ setting goals, positive thinking and attitude/ hoping for the best	Give an example of a character who set goals for themselves and how did they accomplish their goals. Identify a character that had a positive attitude during the film. What are your goals and how do you plan to reach them? How can you be more positive in your life?
O	Overcoming obstacles	Which character struggled during the film and how did he or she overcome their obstacle? Have you ever had to deal with an obstacle in your life, and how did you overcome the obstacle?
S	Strong female characteristics	Give an example of a female character in the film who demonstrated leadership and/ or intelligence. What makes a female a “strong” character? Can you think of a strong female in your life?
I	Importance of helping/ protecting others, friendship	Identify a character in the film that helps or protects someone or is a good friend. Why is it important to help people in need? What makes a good friend?
T	Teamwork	Give an example of teamwork or collaboration in the film. Are you a member of a team? Why is it important to work as a team?
I	Intelligence/ using your mind	Identify a character that used his or her mind to solve a problem. Can you think of an example when you used your mind to solve a problem? Why is school important?
V	Value of leadership/ being a role model	Was there a character that acted as a good leader or role model during the film? Who is your role model? What characteristics made a good leader or role model?
E	Elevate and fight for what you believe in/ doing the right thing	Identify a character who stood up for what he or she believed in. What is something you believe in, that you would fight for?
N	Negativity (living life filled with worry and stress)	Give an example of a character that was worried or stressed. What makes you stressed or worried? What is a good way to deal with your worry or stress?
E	Excessive anger	Identify a character in the film that demonstrates extreme anger. How do you choose to handle anger? How can you be respectful when you are angry?
G	Guns/ knives / explosions / weapons of destruction use	Give an example of a character in the film that uses weapons. What are some consequences of using weapons? Do you know what to do if you find a gun or other weapon?
A	Arguing/verbal altercation	Give an example of a character in the film that argued a lot. If you disagree with someone, what could you do instead of arguing?
T	Taunting/ bullying/ intimidation	Identify a character in the film that uses intimidation towards another character? Have you ever been bullied, or bullied another person? Why is bullying bad? What would you do if you saw someone being bullied?
I	Invincibility/ desire for power/ arrogance	Identify a character in the film that desires invincibility or power. Have you ever used your brain, instead of power, to handle a situation? What bad things can happen to someone who desires power?
V	Violence acts	Give an example of extreme violence or fighting in the film. How does watching violence on TV or in movies make you feel? How can you resolve conflict without fighting or violence?
E	Expressions of disrespect/ poor sportsmanship	Can you think of a character who was disrespectful towards another character? Have you ever shown disrespect or poor sportsmanship towards another person? How did it make that person feel? How could you handle that situation differently?

This study is not without limitations. Although we chose the 25 top-grossing rated-R films released between 2012 and 2017 based on a popular film website, this may in fact not represent the most popular or most viewed films by children and adolescents. Consequently, the demonstrated findings may not be entirely generalizable to rated-R films outside our release date range or those films that did not gain success in box office sales. Additionally, the coding of the films represents a limitation to the study. Inter-viewer variability exists between the reviewers for both positive and negative themes. Despite the fact that coding guidelines were extensively reviewed and decided upon prior to viewing the study films, each reviewer may differ in their interpretation of scenarios, dialogue, and fighting sequences in the study. These differences may be based on several factors such as background, gender, race, and age of the film reviewer. In this study, all reviewers were adults, who may interpret themed events differently than would a member of the pediatric population. Even though our study enacted several categories to assess the severity of violence, our coding system was still unable to assess magnitude within a single category, such as between different types of murder (i.e. via gun or strangling). Nonetheless, although the main objective of this study was to determine the positive and negative themes found in top-grossing rated-R films, the actual number of events may not be as important as the positive and negative themes that are under-represented and require more emphasis in future films. Although the primary goal of this study was to determine specifically the thematic content of the top-grossing rated-R films in our sample, we did not determine the unique beneficial and detrimental effects and roles that exposure to these themes may have on their pediatric audience. 

## Conclusions

In conclusion, based on our sample of rated-R films released between 2012 and 2017, there were twice as many negative themes than positive themes depicted. The most common positive themes were associated with service, collaboration, and honesty/humility, while the most common negative themes were associated with violence. Themes depicted in films can have a profound impact on the development of children and adolescents. With increased access to previously restricted material, children and adolescents may be exposed to more mature and explicit themes at younger ages. As a result we encourage parents/guardians and pediatric health care providers to be cognizant of the reality that their children may be viewing in rated-R films. We recommend co-viewing and active mediation, focusing on the positive and negative themes depicted in rated- R film, as a method to guide children and adolescents through critical periods of their mental and behavioral development.
